# Editorial: Third Window Syndrome

**DOI:** 10.3389/fneur.2021.704095

**Published:** 2021-06-18

**Authors:** P. Ashley Wackym, Yuri Agrawal, Tetsuo Ikezono, Carey D. Balaban

**Affiliations:** ^1^Department of Otolaryngology-Head and Neck Surgery, Rutgers Robert Wood Johnson Medical School, New Brunswick, NJ, United States; ^2^Department of Otolaryngology-Head and Neck Surgery, Johns Hopkins University, Baltimore, MD, United States; ^3^Department of Otorhinolaryngology, Graduate School of Medicine, Saitama Medical University, Saitama, Japan; ^4^Departments of Otolaryngology, Neurobiology, Communication Science & Disorders, Bioengineering and Mechanical Engineering and Materials Science, University of Pittsburgh, Pittsburgh, PA, United States

**Keywords:** cognitive dysfunction, dizziness, perilymph fistula, sound-induced dizziness, superior semicircular canal dehiscence, third window syndrome, vestibular migraine

In this Research Topic, Third Window Syndrome, we brought together recent discoveries of the mechanisms of the associated spectrum of symptoms, dysfunction, novel diagnostic tools and interventions to identify and resolve Third Window Syndrome.

Nearly a century ago, Tullio described the physiologic outcomes of creating a third mobile window in the semicircular canals of pigeons. Since that time, many locations of third mobile windows have been described; however, the sound-induced dizziness and/or nystagmus has been memorialized by the eponym “Tullio phenomenon.” Clinically, the most thoroughly characterized third mobile window is superior semicircular canal dehiscence. In 1998, Minor et al. first reported the diagnosis of CT positive superior semicircular canal dehiscence (SSCD) (1). Minor later reported a conductive hearing loss, which was recognized as a pseudoconductive hearing loss (bone-conduction hyperacusis), as well as a reduced cervical vestibular myogenic potential (cVEMP) threshold in patients with superior semicircular canal dehiscence. While superior semicircular canal dehiscence is well-recognized; it has been reported the existence of a CT negative third window syndrome with the same clinical phenotype of superior semicircular canal dehiscence exists. It has been reported that CT negative third window syndrome is associated with a pseudoconductive hearing loss and an abnormally reduced cVEMP threshold, among other objective findings typically found in superior semicircular canal dehiscence patients. The more general term of Third Window Syndrome has gained acceptance because the same spectrum of symptoms, signs on physical examination and audiological diagnostic findings are encountered with superior semicircular canal dehiscence, cochlea-facial nerve dehiscence, cochlea-internal carotid artery dehiscence, cochlea-internal auditory canal dehiscence, lateral semicircular canal-superior semicircular canal ampulla dehiscence, modiolus, “perilymph fistula,” posterior semicircular canal dehiscence, posterior semicircular canal-jugular bulb dehiscence, superior semicircular canal dehiscence-subarcuate artery dehiscence, superior semicircular canal dehiscence-superior petrosal vein dehiscence, vestibule-middle ear dehiscence, lateral semicircular canal-facial nerve dehiscence, wide vestibular aqueduct in children, post-traumatic hypermobile stapes footplate and in patients with CT negative Third Window Syndrome. A common structural finding in all of these conditions is an otic capsule defect that creates a “third window.”

Over the past 60 years, we have learned much regarding the clinical features, outcomes measured by validated survey instruments and neuropsychology testing as well as objective diagnostic studies in Third Window Syndrome. Beyond the hallmark symptoms of sound-induced otolithic dysfunction (dizziness) and autophony, a wide range of other associated clinical manifestations have been reported, including: cognitive dysfunction, spatial disorientation, anxiety and migraine. The series of papers included in this Research Topic have provided important insights to both scientists and clinicians who deal with these fascinating areas of peripheral vestibular dysfunction and associated pathophysiology.

This Research Topic was truly global in effort and representation with four continents: Asia, Australia/Oceania, Europe, and North America. However, three were not represented: Africa, Antarctica, and South America. There were 15 countries represented: USA; Denmark; Israel; Korea; Germany; Australia; Switzerland; Belgium; Netherlands; Russia; Sweden; England/United Kingdom; New Zealand, Italy; and Japan. There were 118 authors.

In this summary, we highlight the 20 published studies included in this Research Topic and have organized these within the following categories: Diagnostic Studies and New Diagnostic Tools; Cognitive or Spatial Orientation; Health Utility Values; Biomechanics and Pathophysiology; Reviews; Sites of Dehiscence—Rare or Never Before Reported; and Surgical Advances.

## Diagnostic Studies and New Diagnostic Tools

Third Window Syndrome has been an important new clinical diagnosis, and worldwide thousands of patients have benefited from the discovery of this syndrome and the development of successful treatment. Additionally, Third Window Syndrome has also been a very useful pathologic phenomenon with which to better understand the physiology of the vestibular end-organs, and also develop and refine diagnostic tools that probe various elements of the vestibular system. In this Research Topic, several advances in our understanding of vestibular physiology and third window syndrome diagnosis have been reported. Many of these center around the vestibular-evoked myogenic potential (VEMP), which has found primary use in the detection of third mobile window physiology. An overall summary of the diagnostic value of VEMP in Third Window Syndrome has been provided by Noij and Rauch. In their comprehensive synthesis of the topic, the authors highlight that VEMP testing provides an efficient, accurate, and cost-effective screening and diagnostic tool for SSCD physiology, which can then be followed up by temporal bone CT. Noij and Rauch also discuss cutting edge enhancements of VEMP testing, including performing high frequency VEMP testing, which is further explored in detail by several other studies in this Research Topic.

Specifically, Tran et al. reported on the predictive value of using ocular VEMPs elicited at 4 kilohertz (kHz) in the diagnosis of SSCD. The conventional air-conducted sound stimulus for the VEMP test is a 500 Hz tone burst, given that this is thought to be within the physiologic frequency range of the otolith organs. However, the authors find a higher specificity for diagnosing SSCD with the higher frequency sound stimulus, possibly because the otoliths are less sensitive to sound at this higher frequency, and the low impedance system produced by a dehiscence results in the greatest difference in response compared to normal ears at the higher frequencies. Curthoys and Manzari also provide a report underscoring the diagnostic value of the higher frequency 4 kHz stimulus, and offer compelling data that superior canal afferents are activated in a dehiscent inner ear, which respond to 4 kHz stimulation and contribution to the enhancement of this higher frequency response. Curthoys and Manzari also note that performing a single-frequency VEMP has the benefit of reducing the sound exposure and time required for VEMP threshold testing, which requires presentation of multiple sound stimuli at different amplitudes to determine the threshold.

Another study that considers the role of VEMP in SSCD detection is the report from Taylor et al. which found that longer latencies in the bone-conducted ocular VEMP response provide additional diagnostic value in identifying SSCD. The authors provide the intriguing hypotheses that an additional inhibitory node in the stimulation of the inferior oblique muscle in the ocular VEMP response, or collision between ampullopetal and ampullofugal endolymph movement in the membranous canal may cause the delayed response. A final study that considered VEMP and Third Window Syndrome in this Research Topic is the report from Fröhlich et al.. The authors present intriguing data that patients with intracochlear schwannomas have evidence of VEMP abnormalities, including enhancement of VEMP responses. The authors provide the provocative hypothesis that the mass effect of the intracochlear schwannoma may affect inner ear fluid dynamics, and contribute to endolymphatic hydrops, which is some cases can parallel the physiology of Third Window Syndrome.

Additional studies in this Research Topic that have advanced new diagnostic tools include the study by Thai et al. which reported the use of ambient pressure tympanometry in patients with SSCD. Patients were observed to have rhythmic oscillations of their tympanic membranes, consistent with the dehiscence allowing transmission of vascular or cerebrospinal fluid pulsations through the membranous labyrinth, oval window, ossicular chain to the tympanic membrane. Two other studies provided insight into the frequency dynamics of the inner ear fluids. Lee et al. observed that following canal plugging, high frequency vestibular-ocular reflex responses in the plugged canal normalized over the long term, suggesting some preservation of high frequency responses despite resolution of symptoms and closure of the dehiscence. As a corollary, Castellucci et al. reported in a series of 3 patients with labyrinthine fistula that high-frequency responses were attenuated, although the patients still had evidence of canal function given a persistent Hennebert sign, possibly consistent with the preservation of low-frequency responses in a dehiscent state. This set of studies on new diagnostic tools provides evidence that Third Window Syndrome offers a window into the mechanisms of the inner ear!

## Cognitive or Spatial Orientation

As has been noted previously, individuals with peripheral vestibular pathologies including semicircular canal dehiscence have been shown to experience cognitive deficits that improve upon treatment of the vestibular pathology. As such, this Research Topic also explored the theme of cognitive sequelae associated with vestibular impairment. In one article by Wei et al. the link between two commonly-used cognitive outcome measures included in vestibular studies was investigated. Specifically, psychometric paper-and-pencil based cognitive tests and a dynamic test of spatial navigation—the Triangle Completion Task—were compared in ~150 healthy older adults. The study reported that performance on the Triangle Completion Task was significantly associated with scores on tests of visuospatial ability, executive function, and motor processing speed, suggesting that spatial navigation ability taps into the cognitive skills of visuospatial processing, executive function, and motor processing speed. Interestingly, prior research has shown that vestibular function is also related to these cognitive outcome measures. Another study in this Research Topic by Kamil et al. further assessed a quantitative method to capture disoriented spatial navigation behavior—specifically wandering behavior—in a cohort of older adults with Alzheimer disease in whom wandering behaviors are common. Indeed, prior research has shown that patients with Alzheimer have an increased prevalence of vestibular impairment relative to cognitively-intact controls, and that vestibular impairment in turn is associated with spatial cognitive difficulties in Alzheimer patients. The Kamil et al. study established the feasibility of continuous accelerometric monitoring in Alzheimer patients, and provided preliminary data that characteristics of the turning behaviors in Alzheimer patients may identify individuals who wander. Taken together, this series of articles within this Research Topic provided further insight into relevant cognitive outcome measures that can be used in the study of patients with vestibular impairment.

## Health Utility Values

The most common third mobile window producing Third Window Syndrome is SSCD. Patients can experience disabling symptoms and may opt for surgical management. Limited data are available on the impact of SSCD on health-related quality of life (HRQoL) and disease-specific HRQoL more specifically. Ocak et al. performed a prospective analysis on generic HRQoL in SSCD patients compared to healthy age-matched controls. The study participants completed the Health Utility Index (HUI) Mark 2 (HUI2)/Mark 3 (HUI3) questionnaire. For the control group, age-matched participants without otovestibular pathology or other chronic pathology were recruited. The multi-attribute utility function (MAUF) score was calculated for the HUI2 and HUI3. Results of both groups were then compared using the Mann–Whitney *U*-test. For the SSCD case group, the median HUI2 MAUF score was 0.75 and median HUI3 MAUF score was 0.65. For the control group, the median scores were 0.88 and 0.86, respectively. There was a statistically significant difference for both HUI2 (*p* = 0.024) and HUI3 (*p* = 0.011) between the two groups. Not surprisingly, the SSCD patients had a worse generic HRQoL than age-matched healthy controls. Interestingly, one patient with unilateral SSCD had a negative HUI3 MAUF score (−0.07), indicating a health-state worse than death. Ocak et al. concluded that SSCD patients have significantly lower health utility values than an age-matched control group confirms the negative impact of SSCD on generic HRQoL using an instrument that is not designed to be disease-specific but to assess health state in general.

In a study of surgical outcomes in managing Third Window Syndrome caused by cochlea-facial dehiscence, Wackym et al. used the Dizziness Handicap Inventory (DHI) and also the Headache Impact Test (HIT-6) validated survey instruments to assess the changes in scores postoperatively compared to preoperatively for 8 patients who had round window reinforcement surgery for cochlea-facial nerve dehiscence causing third window syndrome and initially and at follow up for 8 patients who elected not to have surgery. The DHI is a 25-item self-assessment inventory designed to evaluate the self-perceived handicapping effects imposed by dizziness/vestibular dysfunction. The HIT-6 is a six-item self-assessment questionnaire used to measure the impact headaches have on a patient's ability to function on the job, at school, at home and in social situations. There was a highly significant improvement in DHI and HIT-6 at pre- vs. postoperative (*p* < 0.0001 and *p* < 0.001, respectively). These findings suggest that round reinforcement surgery for cochlea-facial nerve dehiscence reduces the handicap due to the secondary dizziness/vestibular dysfunction and impact of migraine headaches on their ability to function.

## Biomechanics and Pathophysiology

The current status of conceptual approaches to understanding the biomechanics and pathophysiology of Third Window Syndrome is reviewed and extended in the contributions by Iversen and Rabbitt and by Stenfelt. The former, very approachable review paper provides a comprehensive orientation to biomechanical issues in relation to auditory and vestibular findings. The audiometric results are discussed in the context of a simplified, lumped parameter model of the middle ear and inner ear that will serve well for didactic purposes. The separate sections on oculomotor findings, vestibular-evoked myogenic potentials, and electrocochleography also provide summaries of concepts and the current literature that orient the reader to the state-of-the-art.

The contribution by Stenfelt is an *in silico* study that adapts an earlier model to more deeply probe our formal understanding of basic principles underlying the relative contributions of bone conduction pathways [fluid inertia, middle ear inertial, compression, intracranial (CSF) pressure, and ear canal] to audition in the presence of third window syndromes. The findings predict that fluid inertial effects will have the greatest effect in a simulated semicircular canal dehiscence syndrome and that transmission via cerebrospinal fluid is unlikely to have a significant effect under the same conditions. Secondary predictions regarding the effects of vestibular aqueduct size, amenable to study.

## Reviews

Over the past two decades, advances in diagnostic techniques have raised the awareness of SSCD and treatment approaches have been refined to improve patient outcomes. Eberhard et al. discuss contemporary and emerging diagnostic approaches for patients with Third Window Syndrome due to SSCD, focusing on four challenges: (1) the clinical testing algorithm for quantifying the effects of SSCD; (2) while high-resolution temporal bone CT remains the gold standard for detecting SSCD, a bony defect does not always result in signs and symptoms; (3) even when SSCD repair is indicated, there is a lack of consensus about nomenclature to describe the SSCD, ideal surgical approach, specific repair techniques, and type of materials used; and (4) there is no established algorithm in the evaluation of SSCD patients who fail primary repair and may be candidates for revision surgery. They concluded that comparative outcome studies are needed to assess challenging cases, such as patients with bilateral dehiscence, near dehiscence, revision cases, and concurrent SSCD and migraine disorder. It should be noted that many Third Window Syndrome sites of dehiscence, including SSCD, are associated with migraine headaches and the three variants of migraine (ocular migraine, vestibular migraine, and/or hemiplegic migraine) and are either comorbid, exacerbated by or caused by the otolithic asymmetry producing Third Window Syndrome.

An expanded discussion of Third Window Syndromes and how to distinguish them from PLF is found in the review by Sarna et al. Diagnosing PLFs has been a difficult task ever since their description over a century ago. The authors aimed at providing an update on the classification, diagnosis, and treatment of PLF. New diagnostic criteria are based on the inciting events and confirmation of a specific biomarker and leakage identification plus resolution symptoms after blood patch/surgical plugging. Presently, the novel biomarker cochlin-tomoprotein (CTP) is the best candidate for a specific biomarker, which has been approved by the Japan Ministry of Health, Labor, and Welfare (equivalent agency as the United States Food and Drug Administration [FDA] or the CE Mark for the European Union) for medical diagnosis. Advances in diagnostic criteria, high resolution imaging, and biomarker testing are paving the way for accurate preoperative diagnosis of Third Window Syndrome. The authors concluded that PLF is one of the few etiologies of dizziness, tinnitus, and hearing loss that can be treated surgically. They also emphasized that it is critical to remain vigilant and keep PLF in the differential diagnosis since prompt treatment has the potential to alleviate patients from debilitating vertigo and permanent hearing loss.

## Sites Of Dehiscence—Rare or Never Before Reported

Dasgupta et al. completed a retrospective study of children (aged 5–17 years) diagnosed with rare third window disorders in a tertiary pediatric vestibular unit in the United Kingdom. They investigated the audiovestibular function in these children. The radiographic diagnosis was achieved by high resolution CT scan of the temporal bones. Of 920 children presenting for audiovestibular assessment over a 42 month period, rare third mobile windows were observed in 8 (<1%). These included posterior semicircular canal dehiscence (*n* = 3, 0.3%), posterior semicircular canal thinning (*n* = 2, 0.2%), X linked gusher (modiolus as the third mobile window) (*n* = 2, 0.2%), and a combination of dilated internal auditory meatus/irregular cochlear partition/deficient facial nerve canal (*n* = 1, 0.1%). The majority of these children (87.5%) demonstrated a mixed/conductive hearing loss with an air-bone gap in the presence of normal tympanometry (pseudoconductive hearing loss) in 100% of the children. Transient otoacoustic emissions were absent with a simultaneous cochlear pathology in 50% of the cohort. Features of disequilibrium were observed in 75% and about a third showed deranged vestibular function tests. Video head impulse test abnormalities were detected in 50% localizing to the side of the lesion. Cervical vestibular evoked myogenic potential test abnormalities were observed in all children in the cohort undergoing the test where low thresholds and high amplitudes classically found in third mobile window disorders localized to the side of the defects in 28.5%. In the series, 71.4% also demonstrated absent responses/amplitude asymmetry, some of which did not localize to the side of the third mobile window. Only two children presented with typical third window symptoms. This study suggests that pediatric third window disorders may not present with classical third mobile window features and are variable in their presentations and audiovestibular functions.

The prevalence and distribution of sites of dehiscence in 802 temporal bones of 401 patients with Third Window Syndrome was reported by Wackym et al. However, it should be emphasized that all of their patients had Third Window Syndrome symptoms, whereas the status of Third Window Syndrome symptoms was not reported for the subjects in other published prevalence studies. Wackym et al. identified 463 temporal bones [57.7% (463/802)] with a single site of dehiscence (SSCD, near-SSCD, CT negative Third Window Syndrome, CFD, cochlea-internal auditory canal, wide vestibular aqueduct, lateral semicircular canal, modiolus and posterior semicircular canal, SSCD and superior petrosal sinus, SSCD and subarcuate artery). If the CT negative Third Window Syndrome temporal bones were excluded, there was single site temporal bone dehiscence found in 366 [366/402 (91.0%)]. SSCD and near-SSCD were the most commonly observed site of dehiscence [59% (296/502)]. The second most commonly observed category of radiologic findings in the Third Window Syndrome cohort was CT negative Third Window Syndrome [19.3% (97/502)]. The third most commonly observed site of dehiscence was CFD [10.4% (52/502)]. Regarding multiple sites of dehiscence, there were 38 instances [38/405 (9.38%)] of two site dehiscence (SSCD and CFD, CFD and cochlea-internal auditory canal, CFD and wide vestibular aqueduct, SSCD and cochlea-internal auditory canal, SSCD and posterior semicircular canal-jugular bulb). The combination of SSCD and CFD accounted for 6% (30/502). There was one instance of three sites [3/405 (0.24%)] of dehiscence (SSCD and posterior semicircular canal and wide vestibular aqueduct). The prevalence of multiple-site findings is important to consider when faced with recurrent or incompletely resolved Third Window Syndrome symptoms after plugging a SSCD. In light of their recent observations and the histologic, cadaveric and patient CT scan prevalence of CFD and concurrent SSCD and CFD, they concluded that careful assessment of the presence of CFD in patients with SSCD should be completed and factored into the surgical planning.

The pair of papers contributed by Gadre et al. and Matsuda et al. highlight that acquired or congenital defects in the stapes footplate can create Third Window Syndrome by creating a third mobile window. Gadre et al. reported an observational analytic case studies review of 28 patients (33 ears) managed over an 11-year interval. These patients suffered persistent dizziness following head trauma and demonstrated Tullio phenomena or Hennebert sign. All had neuroradiologists report normal otic capsules on high resolution temporal bone CT scans. However, when the gray-scale invert function was used to visualize the stapes footplate the absence of a normal footplate was evident, thus yielding the preoperative diagnosis. All cases had middle ear exploration to determine if perilymph leakage was present. Intraoperative Valsalva maneuvers were performed to visualize perilymph egress. They performed fat grafting of round and oval windows with none of the patients having deterioration of their hearing. Prior to surgery all patients reported dizziness in response to loud sounds and/or barometric pressure changes. Seven out of 33 ears had demonstrable perilymph leakage into the middle ear; the rest (26 ears) appeared to have membranous or hypermobile stapes footplates. Thirteen patients had a fistula sign positive bilaterally while 15 had unilateral pathology. Twenty-four of the 28 patients (85.7%) showed both subjective and objective improvement following surgery. They concluded that a membranous or hypermobile stapes footplate can occur following head trauma and can cause intractable dizziness typical of Third Window Syndrome.

The contribution by Matsuda et al. reported a patient with a congenital dehiscence of the right stapes footplate. This dehiscence caused long-standing episodic pressure-induced vertigo (Hennebert sign) with intervals of being asymptomatic and normal. At the time of presentation, her increased thoracic pressure changes induced the rupture of the membranous stapes footplate. She had experienced a sudden right-sided hearing loss and severe true rotational vertigo, immediately after nose-blowing. CT scan showed a vestibule pneumolabyrinth. Perilymphatic fistula (PLF) repair surgery was performed. During the operation, a bony defect of 0.5 mm at the center of the right stapes footplate, which was covered by a membranous tissue, and a tear was found in this anomalous membrane. A perilymph-specific protein CTP detection test was positive. The fistula in the footplate was sealed. Postoperatively, the vestibular symptoms resolved, and her hearing improved. A more detailed history revealed that, for 15 years, she experienced true rotational vertigo when she would blow her nose. After she stopped blowing her nose, she would again feel normal. They concluded that this case demonstrated that a congenital defect in the stapes footplate can result in a PLF by seemingly insignificant events such as nose-blowing. Appropriate recognition and treatment of PLF can improve a patient's condition and hence, the quality of life.

## Surgical Advances

Wackym et al. published a series of 16 patients with Third Window Syndrome due to cochlea-facial nerve dehiscence (CFD); 8 who had surgical management [round window reinforcement (RWR)] and 8 who did not. Pre- vs. postoperative Dizziness Handicap Inventory (DHI), Headache Impact Test (HIT-6), and audiometric data were compared statistically. The thresholds and amplitudes for cVEMP in symptomatic ears, ears with CFD and ears without CFD were compared statistically. All 8 in the surgical cohort had a history of trauma before the onset of their symptoms. The mean cVEMP threshold was 75 dB nHL (SD 3.8) for the operated ear and 85.7 dB (SD 10.6) for the unoperated ear. In contrast to superior semicircular canal dehiscence, where most ears have abnormal electrocochleography (ECoG) findings suggestive of endolymphatic hydrops, only 1 of 8 operated CFD ears (1 of 16 ears) had an abnormal ECoG study. The phenotype associated with CFD was typical of the spectrum of signs and symptoms seen in SSCD patients, including 5/16 (31%) who could hear their eyes move or blink and 13/16 (81%) with sound-induced dizziness. Other clinical findings often seen in SSCD were the presence of headache and migraine headaches 15/16 (94%), vestibular migraine with true rotational vertigo episodes 8/16 (50%) and ocular migraine 7/16 (44%); however, ocular migraines were infrequent as were vestibular migraine episodes. The one patient who did not have headaches or migraine headaches had vestibular migraine episodes intermittently. Overall there was a marked and clinically significant improvement in DHI, HIT-6, and Third Window Syndrome symptoms postoperatively for the CFD cohort who had RWR surgery. A statistically significant reduction in cVEMP thresholds was observed in patients with radiographic evidence of CFD. Surgical management with RWR in patients with CFD was associated with improved symptoms and outcomes measures. There was no statistically significant change of hearing in the patients with CFD who underwent RWR. It is emphasized that radiographic CFD is not in itself an indication for surgery and that the most important factor in decision-making should be in the context of clinical symptoms and other diagnostic findings. There are three important presenting symptoms and physical findings that are critical when identifying a Third Window Syndrome, including CFD: (1) sound-induced dizziness; (2) hearing internal sounds; and (3) hearing or feeling low frequency tuning forks in an involved ear when applied to a patient's knee or elbow. Another important observation in the study was that multiple sites of dehiscence in temporal bones with Third Window Syndrome occurs and this finding is important to consider when faced with recurrent or incompletely resolved Third Window Syndrome symptoms after plugging a SSCD.

Mignacco et al. reported a novel strategy in managing a patient with SSCD. While round window reinforcement has been used as a surgical alternative to plugging or resurfacing a SSCD, the authors reported the outcomes of round window reinforcement surgery performed with the application of a Vibrant Soundbridge middle ear implant. The patient experienced recurrent sound-induced vertigo/dizziness, Tullio phenomenon, Hennebert sign, bone conduction hypersensitivity (pseudoconductive hearing loss), and bilateral moderate to severe mixed hearing loss. Cervical vestibular evoked myogenic potentials (cVEMP) and high-resolution CT confirmed bilateral superior semicircular canal dehiscence. The surgical procedure was performed in the right ear as it had worse vestibular and auditory symptoms, a poorer hearing threshold, more evident SSCD by CT and higher amplitude and lower threshold cVEMP findings. With local anesthesia and sedation, round window reinforcement surgery with perichondrium was performed with simultaneous positioning of a Vibrant Soundbridge on the round window niche. At the one and 3 months follow-up after surgery, Vibrant Soundbridge-aided hearing threshold in the right ear improved to mild, and loud sounds no longer elicited either dizziness in the patient.

## Conclusions

In this Editorial, we highlight the 20 published studies included in this Research Topic and organized these in the following categories: Diagnostic Studies and New Diagnostic Tools; Cognitive or Spatial Orientation; Health Utility Values; Biomechanics and Pathophysiology; Reviews; Sites of Dehiscence—Rare or Never Before Reported; and Surgical Advances. The studies of new diagnostic tools provide evidence that Third Window Syndrome offers a window into the mechanisms of the inner ear. Fundamentally there are three important presenting symptoms and physical findings that are critical when identifying a Third Window Syndrome regardless of physical site of the dehiscence: (1) sound-induced dizziness; (2) hearing internal sounds; and (3) hearing or feeling low frequency tuning forks in an involved ear when applied to a patient's knee or elbow. The sound-induced auditory and vestibular activity is distinct from other balance disorders in the sense that the transient vestibular afferent activity is uncoupled from motion of the head or body in space (allocentric reference frame) or from motion of the environment around the head and body (egocentric reference frame). It will also be uncorrelated with contextual visual, somesthetic, and interoceptive sensory information and on-going (or planned) motor activity. Although the studies focused on cognitive and spatial orientation findings in TWS provided further insight into relevant cognitive outcome measures that can be used in the study of patients with vestibular impairments, neither their measures nor validated survey instruments for symptoms (e.g., DHI or HIT-6) are designed to consider the unique features of perceptual incongruities between Third Window Syndrome and other conditions While current tools may be useful for monitoring patient outcomes while managing patients with Third Window Syndrome, there is room for refinement. The biomechanics, pathophysiology and review studies provided useful conceptual and state-of-the-art frameworks to better understand peripheral bases for the signs and symptoms of common forms of Third Window Syndrome. These frameworks are essential for designing specific diagnostic tests and new, potentially therapeutic approaches. Finally, rare and newly identified sites creating a third mobile window were presented and surgical advances to manage various sites resulting in Third Window Syndrome were reported. Together, these 20 publications comprising this Research Topic present an overview of current knowledge and gaps to be filled in our understanding, diagnosis and management of patients with Third Window Syndrome.

## Author Contributions

PAW, YA, TI, and CDB contributed conception, design of the editorial, and wrote sections of the manuscript. PAW, YA, TI, and CDB wrote the first draft of the manuscript. All authors contributed to manuscript revision, then read and approved the submitted version.

## Conflict of Interest

TI holds patents for the test to detect perilymph leakage using the novel biomarker cochlin-tomoprotein (CTP). The remaining authors declare that the research was conducted in the absence of any commercial or financial relationships that could be construed as a potential conflict of interest.
